# Constructing an Invasion Machine: The Rapid Evolution of a Dispersal-Enhancing Phenotype During the Cane Toad Invasion of Australia

**DOI:** 10.1371/journal.pone.0156950

**Published:** 2016-09-22

**Authors:** C. M. Hudson, M. R. McCurry, P. Lundgren, C. R. McHenry, R. Shine

**Affiliations:** 1 School of Life and Environmental Sciences A08, University of Sydney, Sydney, New South Wales 2006, Australia; 2 Anatomy and Developmental Biology, Monash University, Clayton, Victoria 3800, Australia; 3 Geoscience, Museum Victoria, Carlton, Victoria 3001, Australia; 4 Paleobiology, National Museum of Natural History, Smithsonian Institution, Washington, District of Columbia 20560, United States of America; State Museum of Natural History, GERMANY

## Abstract

Biological invasions can induce rapid evolutionary change. As cane toads (*Rhinella marina*) have spread across tropical Australia over an 80-year period, their rate of invasion has increased from around 15 to 60 km per annum. Toads at the invasion front disperse much faster and further than conspecifics from range-core areas, and their offspring inherit that rapid dispersal rate. We investigated morphological changes that have accompanied this dramatic acceleration, by conducting three-dimensional morphometric analyses of toads from both range-core and invasion-front populations. Morphology of heads, limbs, pectoral girdles and pelvic girdles differed significantly between toads from the two areas, ranging from 0.5% to 16.5% difference in mean bone dimensions between populations, with invasion-front toads exhibiting wider forelimbs, narrower hindlimbs and more compact skulls. Those changes plausibly reflect an increased reliance on bounding (multiple short hops in quick succession) rather than separate large leaps. Within an 80-year period, invasive cane toads have converted the basic anuran body plan – which evolved for occasional large leaps to evade predators – into a morphotype better-suited to sustained long-distance travel.

## Introduction

Biological invasions impose profound new evolutionary pressures both upon the invader, and upon the recipient ecosystem [1]. In response to those pressures, organisms can exhibit phenotypic evolution at rates far higher than are usually observed in equilibrial systems [2,3]. For example, individuals at an expanding range edge often exhibit distinctive traits of behavior, physiology and morphology that enhance their rates of dispersal [4,5,6]. The accumulation of dispersal-enhancing traits has been recorded at invasion fronts of organisms as diverse as pine trees (lighter seeds that float further on the wind: [7]), damselflies (larger wing musculature: [8]), birds (larger wings: [9,10]) and rodents (larger feet: [11]; see [12] for a review).

One of the most intensively studied invasions is that of the cane toad (*Rhinella marina*) through tropical Australia [13]. Introduced to northeastern Queensland in 1935 in a futile attempt to control insect pests, toads have spread at an ever-increasing pace (from 1–15 km/yr in the decades post-release, to 55–60 km/yr at present: [14,15]). Radio-tracking studies confirm that range-core toads are sedentary (mean nightly displacement < 10 m) whereas invasion-vanguard toads are highly mobile (> 200 m per night: [16,17,18]). Laboratory-bred offspring raised in common-garden conditions inherit the distinctive dispersal rate [19], dispersal behavior (path straightness: [20]), and immunological functioning [21] of their parents.

Has toad morphology also evolved in ways that facilitate rapid, sustained dispersal? The anuran body plan is highly conservative, and centered around a powerful propulsive system that can allow a frog to leap distances several times its own body length [22]. That spectacular ability has been lost in many anuran lineages, especially fossorial taxa, but they retain the basic anuran morphotype of a large head, a short inflexible spinal column, and a lever system (involving the pelvic girdle and hindlimbs) that allows saltatory locomotion [23]. In cane toads, invasion-vanguard toads were reported to have longer hindlimbs relative to body length than did the toads a year behind the front; and those longer legs were associated with more rapid dispersal (from radio-tracking: [17,24]) but also, with vulnerability to spinal arthritis [25,26]. Given the functional integration of body components, and the potential influence of many phenotypic traits on locomotor speed and endurance, we speculated that other morphological features might well have evolved also during the course of the toads’ Australian invasion. Accordingly, we conducted Computerized X-ray Tomography (CT) scanning of toads to examine whether skeletal morphology varies between the long-colonised (eastern) and recently invaded (western) extremes of the species’ current distribution in Australia.

## Results

**Fig 1:**
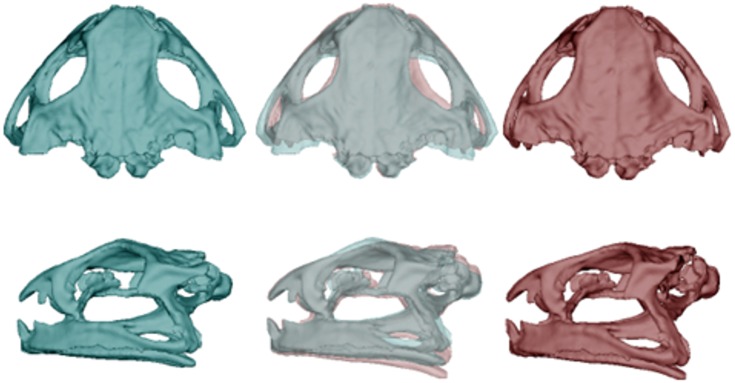
Differences in morphology of the skull between populations of cane toads, based on analyses of 52 specimens. Dorsal and lateral views depict mean skull morphology of toads from long-colonised areas (left, blue) and those from invasion-front populations (right, red). The central images overlay the ones on either side to reveal points of divergence, in this case reflecting the transformation from a low (-0.06) to high (0.06) PC1 score.

**Fig 2:**
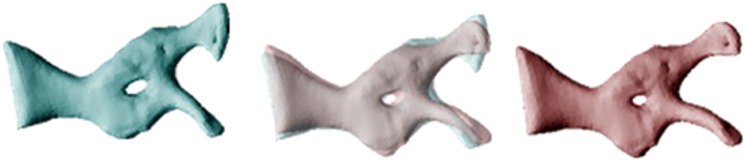
Differences in morphology of the pectoral girdle between cane toads from long-colonised (left, blue) and invasion-front populations (right, red) for 22 specimens. The central image overlays the ones on either side to reveal points of divergence, in this case reflecting a transformation from a low (-0.09) to high (0.06) PC2 score, and a low (-0.06) to high (0.06) PC3 score.

**Fig 3:**
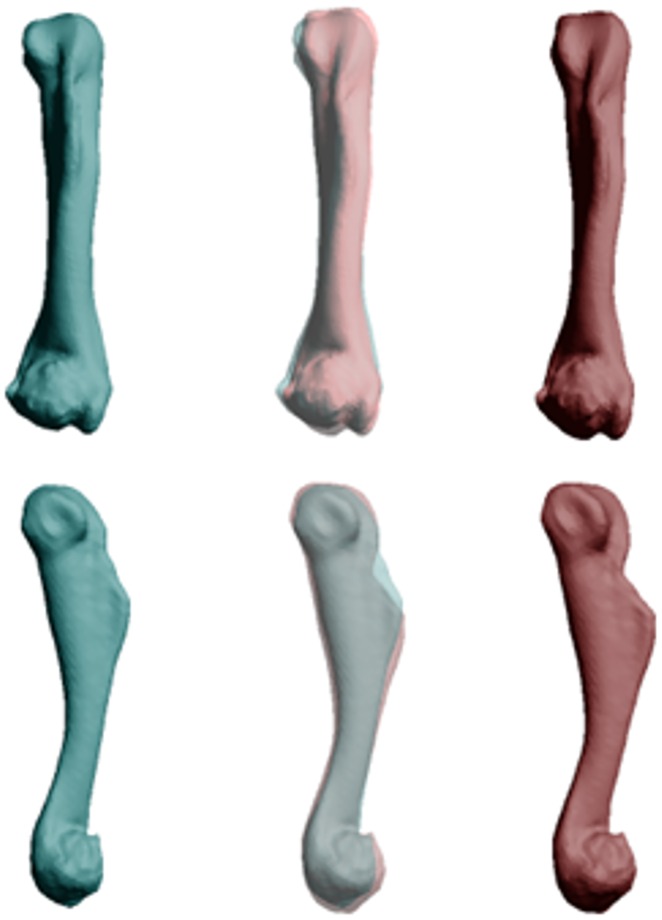
Differences in morphology of the humerus between populations of cane toads based on scans of 19 specimens (10 QLD, 9 WA). The images show mean values for cane toads from long-colonised (left, blue) and invasion-front populations (right, red). The central image overlays the ones on either side to reveal points of divergence, in this case reflecting a transformation from a low (-0.04) to high (0.04) PC1 score.

**Fig 4:**
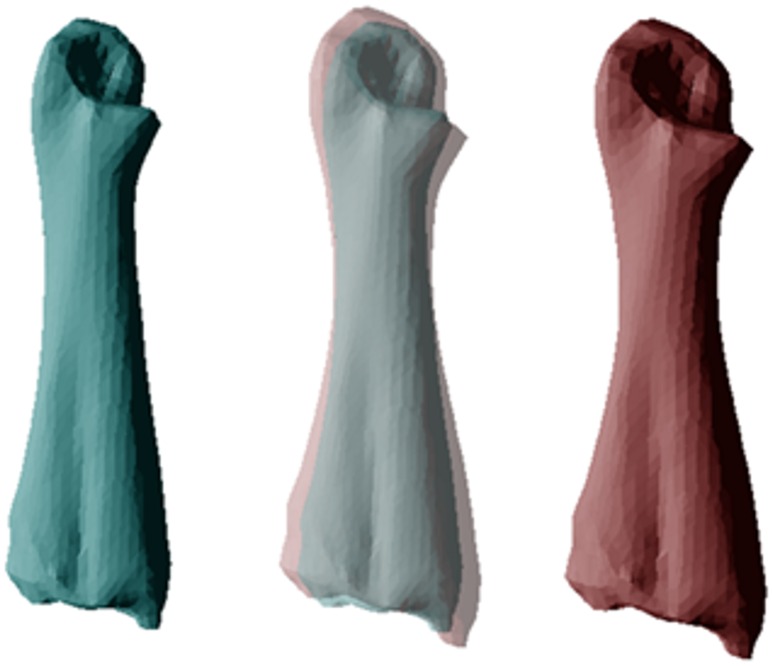
Differences in morphology of the radioulna between cane toads from two populations based on scans of 21 specimens (11 QLD, 10 WA). Mean values for cane toads from long-colonised are shown on the left (in blue) and means for invasion-front populations on the right (in red). The central image overlays the ones on either side to reveal points of divergence, in this case reflecting a transformation from a high (0.04) to low (-0.04) PC3 score, and a low (-0.04) to high (0.02) PC4 score.

**Fig 5:**
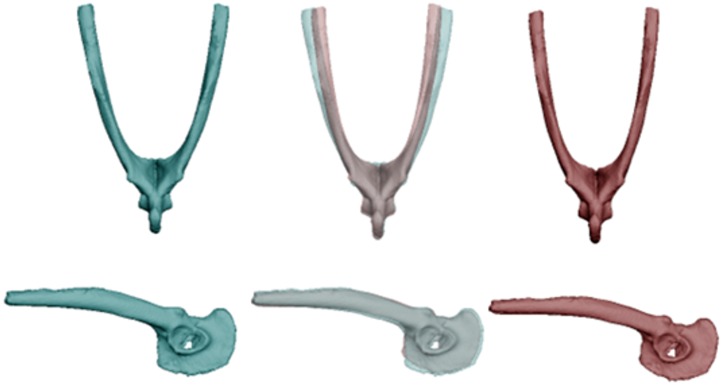
Differences in morphology of the pelvic girdle between cane toads from two regions, based on scans of 35 specimens (20 QLD, 15 WA). Dorsal and lateral views depict changes to mean pelvis morphology between toads from long-colonised areas (left, blue) and those from invasion-front populations (right, red). The central image overlays the ones on either side to reveal points of divergence. These images depict the transformation from a high (0.04) to low (-0.04) PC3 score, and a high (0.03) to low (-0.02) PC6 scoren.

**Fig 6:**
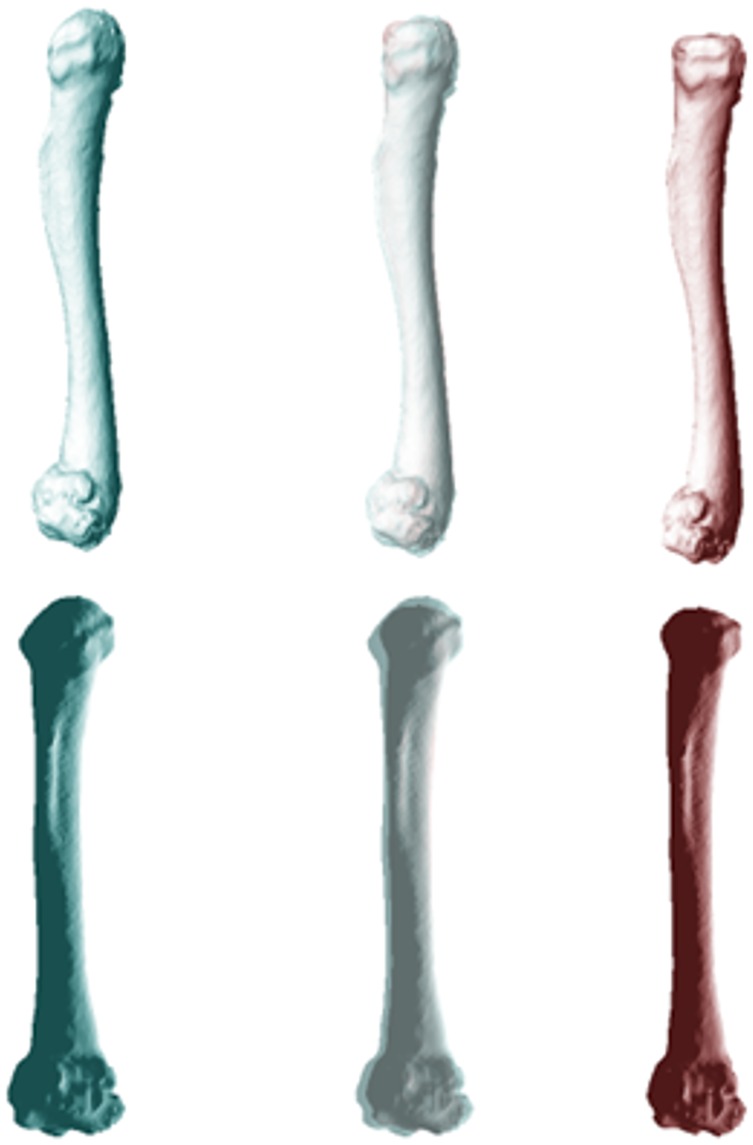
Differences in morphology of the femur between populations of cane toads for 36 specimens (20 QLD, 16 WA). Mean values for long-colonised colonised populations are shown on the left (in blue) and those from invasion-front populations on the right (in red). The central images overlay the ones on either side to reveal points of divergence. These images depict the transformation from a high (0.04) to low (-0.04) PC3 score.

**Fig 7:**
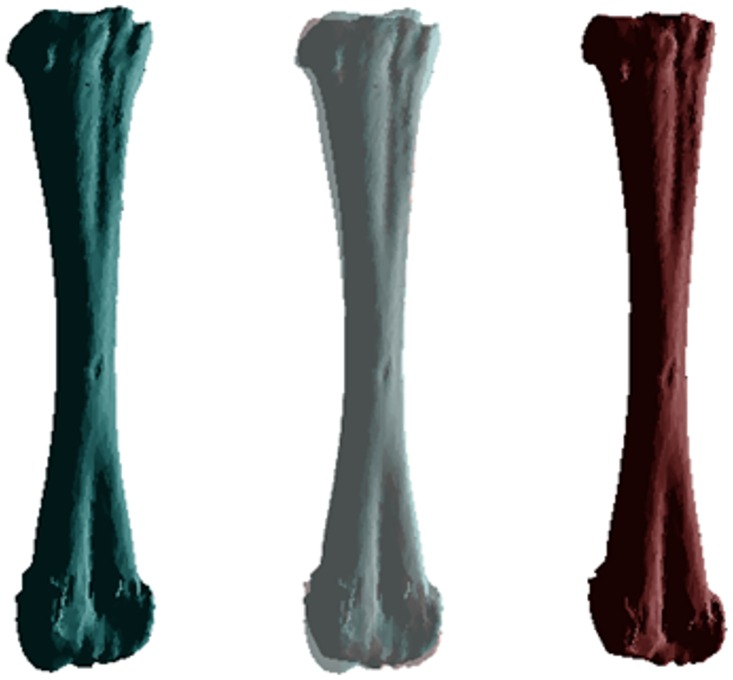
Differences in morphology of the tibiofibula between cane toads from long-colonised (left, blue) to invasion-front populations (right, red), based on 35 specimens (20 QLD, 15 WA). The central image overlays the ones on either side to reveal points of divergence. These images depict the transformation from a low (-0.04) to high (0.04) PC2 score, and a high (0.02) to low (-0.02) PC5 score.

The two populations did not differ significantly in mean SVL (108.5 vs 102.2 mm in WA and QLD respectively; F_1,53_ = 3.43, p = 0.07), but differed strongly in morphology. MANOVAs on each osteological element except the suprascapula detected significant differences between populations (Table 1). For each bone, post-hoc one-way ANOVAs detected at least one PC axis differing significantly between toads from the two areas (Table 2; and see Table I in S1 file for detailed descriptions of the influence of each PC axis on bone morphology). Compared to range-core conspecifics, invasion-front cane toads had dorso-ventrally deeper skulls (+4.5%) with a wider inter-orbital distance (+6.3%; Fig 1), and pectoral girdles that were more curved (+2.5–7.2%), with wider articulation surfaces at the gleohumoral joint (+7.5–16.5%; Fig 2). The humerus and radioulna of invasion-front toads were larger at the elbow (+2.7–5%; Figs 3 and 4), and the humerus was straighter and longer (+9.2%; Fig 3), whereas the radioulna was wider and less sharply angled at the ulnar end of the wrist (Fig 4). Invasion-front toads had a narrower pelvis (-0.5%), with a smaller pelvic area (-4.5%; Fig 5), and smaller heads on both the femur (-2.8–5.4%; Fig 6) and tibiofibula (-2.5–9.4%; Fig 7), with a decrease in total femur length (-8.1%; Fig 6). The tibiofibula also was larger at the knee, but smaller at the ankle, in invasion-front individuals, creating a difference in total length (-12.5%; Fig 7). In summary, as cane toads have invaded across tropical Australia they have evolved substantial changes in skeletal morphology (more robust forelimbs, less robust hindlimbs, changes to the pectoral and pelvic girdles, and a narrower skull).

**Table 1:** Geographic divergence in shapes of bones in cane toads from western (invasion-front) and eastern (range-core) populations in Australia. The table shows MANOVA results from the first six principal component axes for each bone. Bone elements that are significantly different in shape between toads from WA versus QLD are highlighted in boldface.

Bone	F-value	DF	P-value
Skull	1.36	6,45	**<.0001**
Pectoral girdle	2.56	6,15	**0.002**
Suprascapula	0.98	6,16	0.058
Humerus	2.07	6,12	**0.018**
Radioulna	3.00	6,14	**0.001**
Pelvic girdle	0.86	6,28	**0.005**
Femur	0.63	6,29	**0.02**
Tibiofibula	0.74	6,28	**0.011**

**Table 2:** Principal Components representing statistically significant (P < 0.05) morphological divergences in shape between invasion-front (WA) and range-core (QLD) populations of cane toads. The larger mean value for each PC is highlighted in boldface font. Ranking of axes (in terms of variance explained) is calculated separately for each bone.

Bone	PC	% variance	QLD mean	WA mean	P-value
Skull	1	29.3	-0.01205	**0.01205**	0.004
Pectoral girdle	2	15.2	-0.01590	**0.01590**	0.004
Pectoral girdle	3	12.8	-0.01067	**0.01067**	0.045
Humerus	1	20.9	-0.01159	**0.01288**	0.018
Radioulna	3	11.8	**0.00988**	-0.01086	0.005
Radioulna	4	8.0	-0.00624	**0.00687**	0.041
Pelvic girdle	3	12.9	**0.00473**	-0.00361	0.018
Pelvic girdle	6	5.8	**0.00271**	-0.00361	0.046
Femur	3	8.8	**0.00631**	-0.00789	0.002
Tibiofibula	2	11.5	-0.00407	**0.00542**	0.014
Tibiofibula	5	7.0	**0.00318**	-0.00424	0.013

## Discussion

The rapid evolution of a high-dispersal phenotype of cane toads in Australia has been achieved via a remarkable divergence in skeletal morphology between individual toads from invasion-front versus range-core populations. These substantial changes (e.g. a 9.2% increase in humerus length and 12.5% decrease in tibiofibula length) represent rapid phenotypic evolution, as they have occurred over an 80-year period, within the span of a human lifetime. Even more remarkably, those changes have occurred within a body plan that is otherwise highly conservative, not just within the > 500 species of the Family Bufonidae [27], but even within the > 6,500 species of anurans worldwide [22]. Major adaptive radiations into distinctive niches (arboreal, aquatic, or fossorial ecotypes) have been associated with changes in overall anuran shape (especially of limb proportions: [28]), but distantly-related anurans with similar ecological niches exhibit extensive morphological similarities [29,30]. Indeed, that conservatism has been a major obstacle to phylogenetic analyses based on morphology [31].

The Bufonidae rapidly achieved a near-global distribution after originating in South America and colonizing North America, Eurasia and Africa between 78 to 98 Ma [32]. This range expansion was primarily accomplished by toad species phenotypically similar to the cane toad [33]. During its expansion across Australia, the cane toad has further elaborated these dispersal-enhancing morphological modifications. Bufonids are more capable of sustained locomotion than are most other anurans, due to cardiovascular systems that can supply oxygen to active tissues over long periods [34,35,36,37]. Cane toads at the invasion front have been reported to show greater endurance than do conspecifics from range-core areas [38]; but see [39], plausibly reflecting selection on this trait at the invasion front. To transform the bufonid body plan into a long-distance disperser, the other major changes required are to the locomotor apparatus.

Unlike other saltatory anurans that rely on maximizing jump distance to escape predators [23], toad locomotion involves a combination of crawling and hopping [40]. Although their maximal jump distances are lower than those of many similarly-sized anurans, toads have evolved to use their forearms to absorb the shock of landing [41]. That role of the forearms has been expanded to support a novel locomotor mode that involves a cyclical hopping gait (hereafter, “bounding”) for rapid, sustained locomotion [42,43]. By eliminating the pause between successive leaps, a bounding toad can utilize the stored energy from compression of the limbs upon landing, to power the subsequent bound [43].

Our data show that cane toads in the invasion vanguard exhibit larger forearms (especially, wider joints), and smaller hindlimbs, with corresponding alterations to the pectoral and pelvic girdles. These changes suggest that toads at the invasion-front rely more on their forearms during dispersal—consistent with the biomechanical demands of sustained, cyclical hops. The morphological changes that have occurred over the course of the toads’ invasion have produced wider forearms (better able to absorb shock on landing) and a reduction in hindlimb power (to facilitate shorter bounds, rather than huge leaps). Although we have no data on the stresses imposed by the formidable athletic achievements of invasion-front toads, the high incidence of spinal arthritis in such animals [25,26] hints that the changes we have recorded may include adaptations to reduce such stress (as well as to increase the energy efficiency or velocity of locomotion). The apparent contradiction between our results and those of [3] (decrease *versus* increase in relative hindlimb length) are due to curvilinearities in this trait. Hindlimb length has decreased overall during the toad’s Australian invasion (current study), but is higher at the invasion front than in less-recently-colonised areas (unpubl. data).

The changes in skull shape are more difficult to interpret. Although the skull is not usually considered as a component of the locomotor system, the degree of facial tilt in Leporids (Mammalia, Lagomorpha) correlates with locomotor mode, perhaps because changes to cranial structure can increase the visual field of the organism [44]. The shift in cranial morphology in between QLD and WA toads may reflect an advantage of visual awareness in completing multiple rapid hopping and landing cycles. The increase in cranial height (plus the lateral skull compression in invasion-front toads) also may reduce the risk of injury to the brain from repeated take-offs and landings.

In the eighty years following their introduction to Australia, cane toads have expanded their range to an area greater than 1.2 million km^2^ [14]. This expansion has occurred at an increasing rate, with the invasion front advancing more rapidly each year post-colonisation [14,15]. In the process of evolving a rapid-dispersal phenotype, Australian *R*. *marina* have undergone substantial changes in skeletal morphology. Those changes may have arisen either through natural selection (because faster dispersal enables individuals to exploit resource-rich areas before competitors arrive: [45] and/or spatial sorting (wherein traits that accelerate dispersal accumulate at an expanding range edge, regardless of fitness consequences: [46]). In the course of their Australian invasion, cane toads are not only changing the rate at which they move, but the way that they move as well. The distinctive morphology of the invasion-front toads suggests that they have shifted from a sedentary lifestyle that requires occasional hops, to one where they migrate westward by rapid, repeated bounding. Although skeletal morphology is conservative across anurans, the intense pressures stimulated in a biological invasion can rapidly sculpt an organism’s morphology, as well as its physiology and behavior, in ways that enable it to move further and faster than its ancestors.

## Materials and Methods

### Study species and collection sites

This study was conducted with approval from the Animal Care and Ethics Committee of the University of Sydney (6705). Toads were euthanized via lethabarb injections. Between October and December, 2013 we collected 30 toads from two recently invaded populations in Western Australia; El Questro Home Valley Station (16°0′S, 127°58′E) and Kununurra (15°46′S, 128°44′E). We also obtained 30 toads from a long-colonised population in Townsville, Queensland (19°15′S, 146°49′E). The western sites were colonised by toads in 2012 (El Questro) and 2010 (Kununurra; [47]) while the eastern site was invaded in 1940 [16], soon after toads were imported to Australia. Following capture, these animals were humanely euthanized and shipped to Melbourne, VIC for imaging. We collected adults of both sexes, as well as juveniles to capture a range of body sizes for each population.

### Imaging and post-processing

From the initial 60 toads collected, 55 (QLD n = 27, WA n = 28) were used for scanning and geometric morphometric analysis (Qld, 16 males, 11 females, range 93.1 to 119.4 mm snout-vent length [SVL]; WA 13 males, 7 females, 8 not sexed, range 72.7 to 125.1 mm SVL). The toads were scanned at Melbourne Brain Center using a Siemens 128 slice Computerised X-ray Tomography (CT) system. The resulting image stacks were imported into Mimics V16 software for data segmentation. Within each scan, each anatomical feature was digitally isolated and exported in polygon file format.

### Landmarks and geometric morphometric analysis

Landmarks were recorded using Landmark (version 3.0.0.6) software [48] as three-dimensional Cartesian co-ordinates on the surface meshes. Figures and descriptions of the landmark locations are detailed in Figures A–H and Tables A–H in S1 File. To eliminate size differences between individuals and to correct the dataset for translation and rotation we conducted a generalized Procrustes analysis in Morphologika (version 2.5; [49]), followed by a principal components analysis (PCA) to examine variation in shape. The first six principal components (PC) of each element were compared between populations using MANOVAs. Following this, each PC was then compared between populations and sexes with a one-way ANOVA. Significant PCs were also tested against centroid size (lnCS) to remove the effect of ontogeny on bone morphology. PCs where shape variation was more strongly linked to centroid size than to geographic origin were excluded. Sexual dimorphism was minor, accounting for less than 5% of variation in shape. We excluded PCs that were sexually dimorphic from comparisons between the populations, to avoid sample sex ratios confounding comparisons among areas. For simplicity, we report results from one-factor ANOVAs with area of origin as the factor, combining data from both sexes. For each PC axis that differed significantly between toads from eastern *versus* western Australia, we produced visualizations of mean shape variation for invasion-front and range-core individuals using EVAN toolbox V2.1 (Figs 1 to 7). Throughout this manuscript, figures containing visualizations were created using a hypothetical long-colonised toad as the reference (derived from the population mean shape), and a hypothetical invasion-front toad as the target shape. Visualizations reflect the shift in morphology from eastern to western toads.

To produce simplified estimates of the magnitude of difference between significant PC values we compared the three-dimensional Cartesian co-ordinates from Landmark (version 3.0.0.6) for specific regions of interest (e.g. total humerus length, points 1 and 6) for each individual, after correcting for overall size. Using the Pythagorean Theorem: distance^2^ = (x_2_-x_1_)^2^ + (y_2_-y_1_)^2^ + (z_2_-z_1_)^2^ we calculated linear distances between points, and estimated the percent change in size between population means.

## Supporting Information

S1 File Landmarks and descriptions for bone elements examined in this study: Skull (Figure A, Table A), pectoral girdle (Figure B. Table B), suprascapula (Figure C, Table C), humerus (Figure D, Table D), radioulna (Figure E, Table E), pelvic girdle (Figure F, Table F), femur (Figure G, Table G), and tibiofibula (Figure H, Table H). Also included are detailed descriptions of the effect of a large mean value on bone shape for significant PC axes (Table I).
(DOCX)
