# Supplementary material for: Constructing an Invasion Machine: The Rapid Evolution of a Dispersal-Enhancing Phenotype During the Cane Toad Invasion of Australia
Source: PLoS One. 2016 Sep 22;11(9):e0156950. doi: 10.1371/journal.pone.0156950 (PMC5033235; doi:10.1371/journal.pone.0156950)
Supplement: S1 File — Also included are detailed descriptions of the effect of a large mean value on bone shape for significant PC axes (Table I). (DOCX) [file pone.0156950.s001.docx]

S1 Fig A and Table A

| Skull Landmarks | |
| --- | --- |
| 1 | Occipital condyle |
| 3 | Posterior squamosal process |
| 5 | Posterior edge of quadratojugal bone |
| 7 | Anterior squamosal process (next to orbit) |
| 9 | Anterior edge of fossa between pterygoid and quadratojugal |
| 11 | Widest point of curve on fronto-parietal bone |
| 13 | Upper curve of nasal bone (dorsal) |
| 15 | Lower curve of nasal bone (dorsal) |
| 17 | Terminal edge of premaxillary bone |
| 19 | Top of dentary bone |


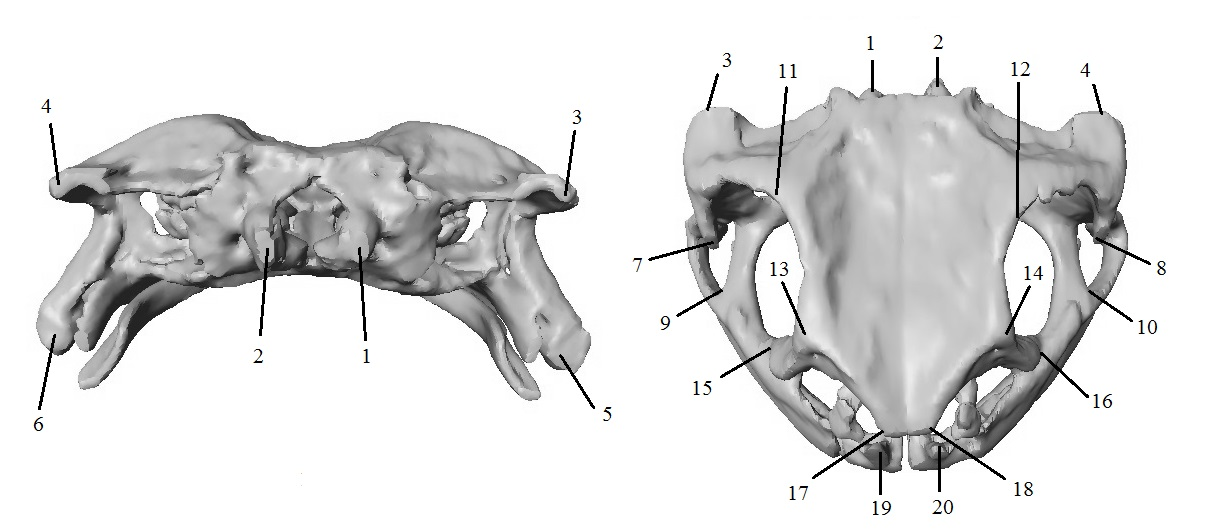


S1 Fig B and Table B


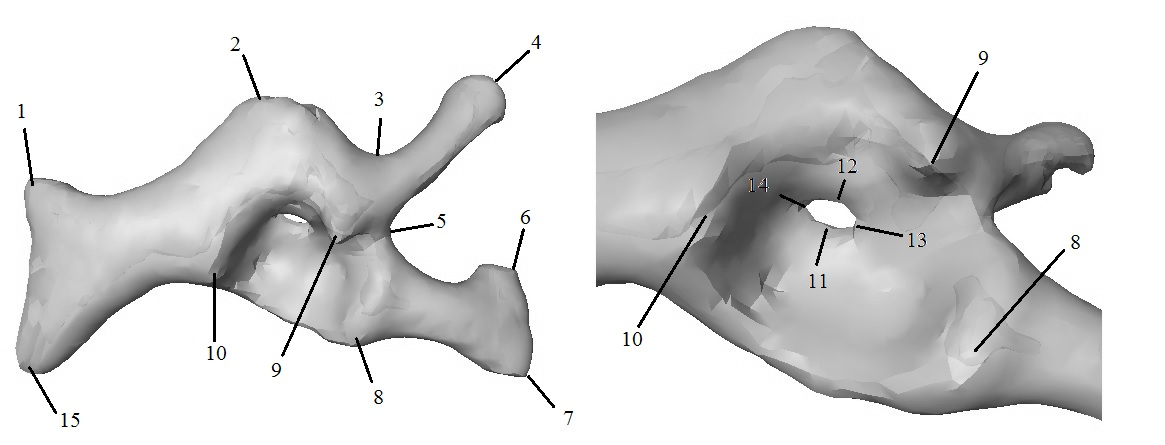


| Pectoral Girdle Landmarks | |
| --- | --- |
| 1 | (Scapula) Anterior articulation point with suprascapula |
| 2 | Highest point on connecting cartilage between clavicle and scapula |
| 3 | Lowest point in groove of clavicle |
| 4 | Terminal end of clavical (sternoclavicular joint) |
| 5 | Mid-point on groove between clavicle and coracoid |
| 6 | Anterior process of coracoid (articulation point with sternum) |
| 7 | Posterior process of coracoid (articulation point with sternum) |
| 8 | Proximal ridge of glenohumeral joint |
| 9 | Medial ridge of glenohumeral joint |
| 10 | Distal ridge of glenohumeral joint |
| 11 | Dorsal aperture of glenohumeral fossa |
| 12 | Ventral aperture of glenohumeral fossa |
| 13 | Distal aperture of glenohumeral fossa |
| 14 | Proximal aperture of glenohumeral fossa |
| 15 | (Scapula) Posterior articulation point with suprascapula |

S1 Fig C and Table C


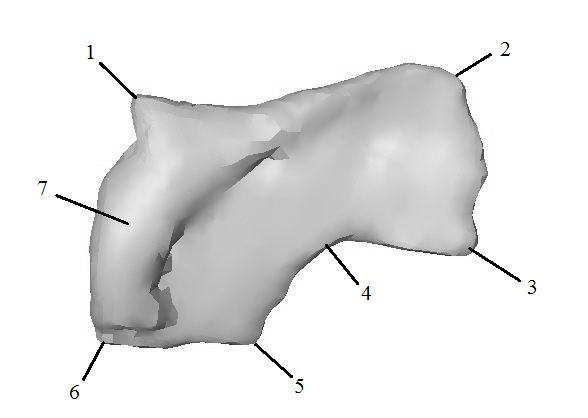


| Suprascapular Landmarks | |
| --- | --- |
| 1 | Anterior distal edge, at articulation point with scapula |
| 2 | Anterior proximal edge of bone |
| 3 | Posterior proximal edge of bone |
| 4 | Mid-point of curvature between points #3 and #4 |
| 5 | Posterior distal edge – opposite point #6 |
| 6 | Posterior distal edge, at articulation point with scapula |
| 7 | Highest point on cleithrum |

S1 Fig D and Table D


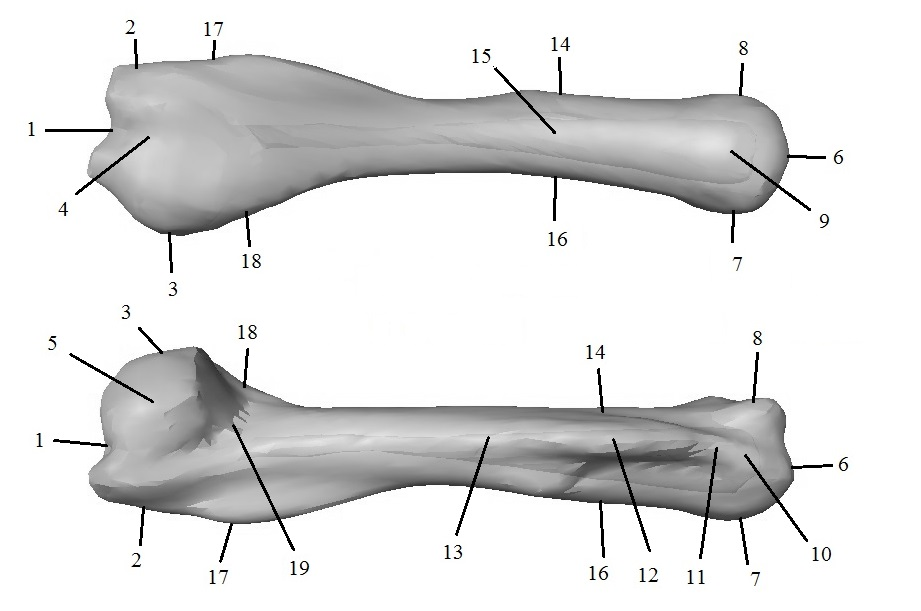


| Humerus Landmarks | |
| --- | --- |
| 1 | Terminal end of posterior extremity (cubital joint) |
| 2 | Lateral edge of posterior extremity (trochlear side) |
| 3 | Opposite point #2 (non-trochlear side) |
| 4 | Doral edge of posterior extremity |
| 5 | Opposite point #4 (ventral) |
| 6 | Terminal end of anterior extremity (glenohumeral joint) |
| 7 | Lateral edge of anterior extremity (non-trochlear side) |
| 8 | Opposite point #7 (trochlear side) |
| 9 | Dorsal edge of anterior extremity |
| 10 | Opposite point #9 (ventral) |
| 11 | Anterior edge of crista deltoidea |
| 12 | Mid-point of crista deltoidea |
| 13 | Posterior edge of crista deltoidea |
| 14 | Lateral mid-point of bone (non-trochlear side) |
| 15 | Opposite point #12 |
| 16 | Opposite point #14 |
| 17 | Lateral edge of crista medialis (where it meets head of humerus) |
| 18 | Opposite point # 17 |
| 19 | Groove under head at cubital joint |


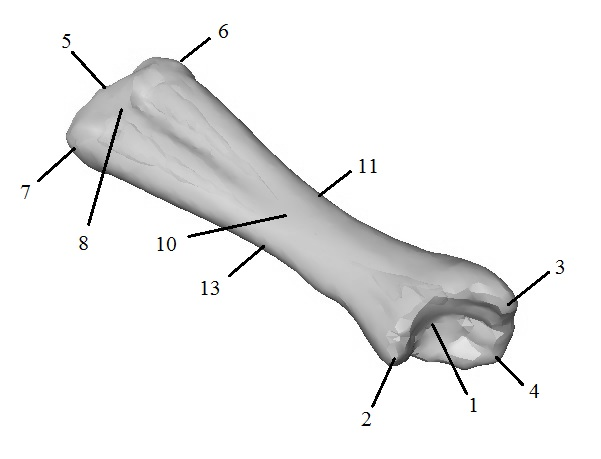
S1 Fig E and Table E

| Radioulna Landmarks | |
| --- | --- |
| 1 | Centre of greater sigmoid cavity (cubital joint) |
| 2 | Capitulum radii |
| 3 | Lower ridge of olecranon |
| 4 | Upper ridge of olecranon |
| 5 | Terminal end, at groove between ulna and radius (radiocarpal joint) |
| 6 | Lateral (ulnar) terminal end |
| 7 | Lateral (radial) terminal end |
| 8 | Dorsal terminal end |
| 9 | Ventral terminal end |
| 10 | Dorsal midpoint of bone |
| 11 | Lateral (ulnar) midpoint |
| 12 | Opposite point #10 (ventral) |
| 13 | Opposite point #11 (radial) |

S1 Fig F and Table F


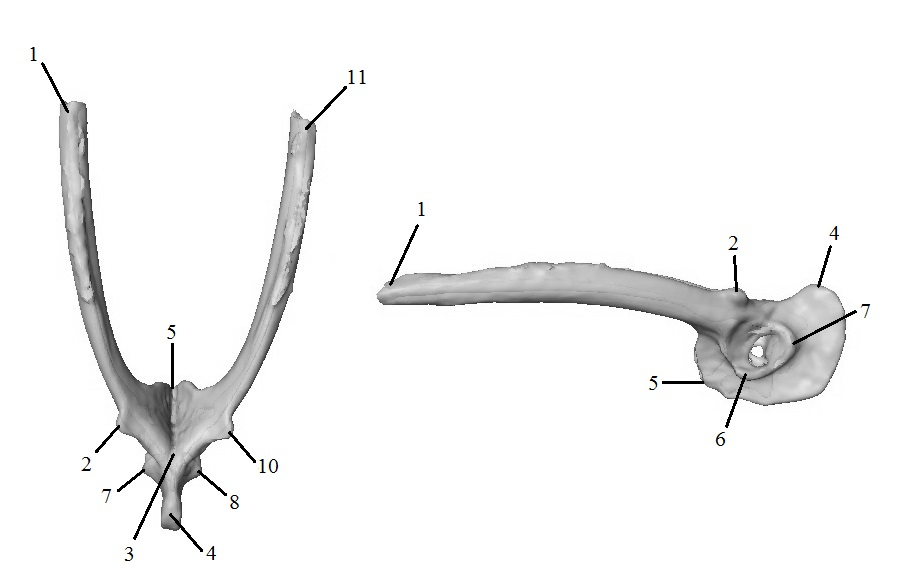


| Pelvic Girdle Landmarks | |
| --- | --- |
| 1 | Terminal point of left iliac arm |
| 2 | Superior process of left ilium |
| 3 | Meeting point between iliac arms |
| 4 | Tuber ischii |
| 5 | Inferior process of the ilium |
| 6 | Left ischial acetabular margin |
| 7 | Left pubic acetabular margin |
| 8 | Right ischial acetabular margin |
| 9 | Right pubic acetabular margin |
| 10 | Superior process of right ilium |
| 11 | Terminal point of right iliac arm |

S1 Fig G and Table G


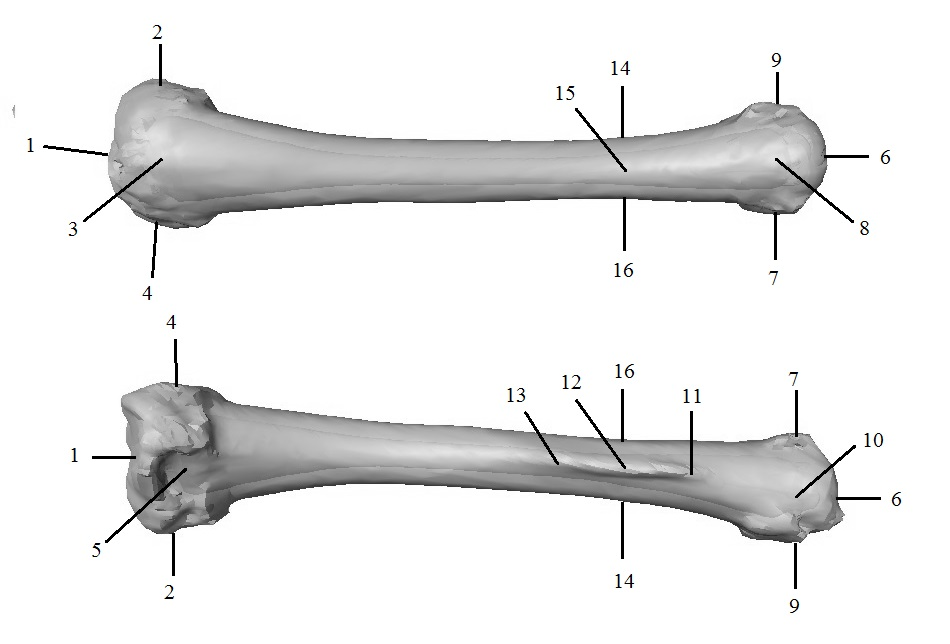


| Femur Landmarks | |
| --- | --- |
| 1 | Terminal end of posterior extremity (patellofemoral joint) |
| 2 | Lateral end of posterior extremity (left side) |
| 3 | Opposite point #3 |
| 4 | Dorsal end of posterior extremity (top of knee) |
| 5 | Opposite point #5 (ventral) |
| 6 | Terminal end of anterior extremity (acetabular joint) |
| 7 | Lateral end of anterior extremity (right side) |
| 8 | Opposite point #7 |
| 9 | Dorsal end of anterior extremity |
| 10 | Opposite point #9 (ventral) |
| 11 | Anterior edge of crista femoralis |
| 12 | Mid-point of crista femoralis |
| 13 | Posterior edge of crista femoralis |
| 14 | Lateral mid-point of bone (right side) |
| 15 | Opposite point #12 (dorsal) |
| 16 | Opposite point #14 |

S1 Fig H and Table H


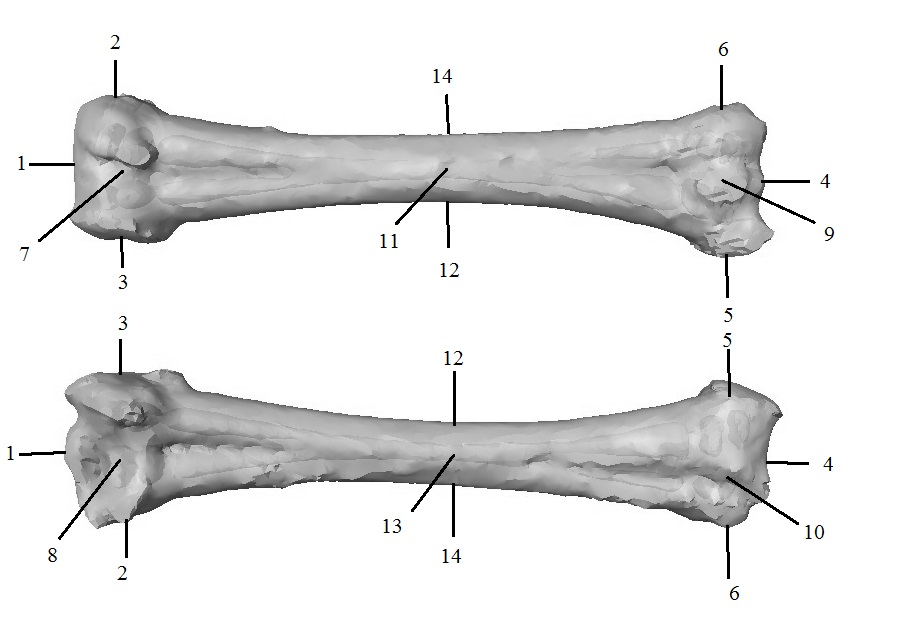


| Tibiofibula Landmarks | |
| --- | --- |
| 1 | Terminal edge of anterior extremity (patellofemoral joint) |
| 2 | Lateral end of anterior extremity (left side) |
| 3 | Opposite point #2 |
| 4 | Terminal edge of posterior extremity (articulation point with tarsus) |
| 5 | Lateral edge of posterior extremity (same side as calcaneum) |
| 6 | Opposite point #5 (same side as astragalus) |
| 7 | Dorsal end of anterior extremity |
| 8 | Opposite point #7 (inside of knee) |
| 9 | Dorsal end of posterior extremity (inside of ankle) |
| 10 | Opposite point #9 |
| 11 | Mid-point of bone (outside edge) |
| 12 | Lateral midpoint (right side) |
| 13 | Opposite point #11 (inside edge) |
| 14 | Opposite point #12 |

S1 Table I

| **Bone** | **PC Axis** | **Description (of large PC value)** |
| --- | --- | --- |
| Skull | 1 | Increased cranial height, shortening of distance between posterior squamosal processes (points 3 and 4) and fronto-parietal curve above orbit (points 11 and 12), resulting in lateral skull compression. |
| Pectoral girdle | 2 | Increased aperture of glenohumoral fossa (points 11-14). Decreased height of connecting cartilage between clavicle and scapula (point 2). |
| Pectoral girdle | 3 | Increased curvature of pectoral girdle, larger glenohumoral joint (points 8, 9 and 10). |
| Humerus | 1 | Increased bone straightness (angle between points 1 and 6). Larger head of bone at cubital joint (increased distance between points 4 and 5). |
| Radioulna | 3 | Compression of cubital joint (decreased distance between 2, 3 and 4). Increased height of bone at radiocarpal joint (points 8 and 9). |
| Radioulna | 4 | Increased distance between points 2 and 4 (at cubial joint). Ulnar end of radiocarpal joint (point 6) less angular, resulting in flattening at wrist. |
| Pelvic girdle | 3 | Increased distance and angle between iliac arms (points 1, 11). Inferior process of ilium upturned (decreased distance between points 3 and 5). |
| Pelvic girdle | 6 | Broadening of acetabulum, increased height of superior iliac processes (points 2, 10), decreased height of tuber ischium. |
| Femur | 3 | Heads of bone larger at patellofemoral (points 1-5) and acetabular joints (points 6-10) |
| Tibiofibula | 2 | Heads of bone smaller at patellofemoral (points 1,2,3,7,8) and tarsal joint (points 4,5,6,9,10). |
| Tibiofibula | 5 | Reduced size of patellofemoral head, expressed as increased distance of point 1 from midpoint |
